# Differential effect of corn oil-based low *trans *structured fat on the plasma and hepatic lipid profile in an atherogenic mouse model: comparison to hydrogenated *trans *fat

**DOI:** 10.1186/1476-511X-10-15

**Published:** 2011-01-20

**Authors:** Yun-Young Cho, Eun-Young Kwon, Hye-Jin Kim, Seon-Min Jeon, Ki-Teak Lee, Myung-Sook Choi

**Affiliations:** 1 Department of Food Science and Nutrition, Kyungpook National University, Daegu, South Korea; 2 Food and Nutritional Genomics Research Center, Kyungpook National University, Daegu, South Korea; 3 Department of Food Science and Technology, Chungnam National University, Daejeon; 4 Food R&D, CJ Cheiljedang Corp., Seoul, South Korea

## Abstract

**Background:**

*Trans *fat are not desirable in many aspects on health maintenance. Low *trans *structured fats have been reported to be relatively more safe than *trans *fats.

**Methods:**

We examined the effects of low *trans *structured fat from corn oil (LC), compared with high *trans *fat shortening, on cholesterol and fatty acid metabolism in apo E deficient mice which is an atherogenic animal model. The animals were fed a high *trans *fat (10% fat: commercial shortening (CS)) or a low *trans *fat (LC) diet for 12 weeks.

**Results:**

LC decreased apo B and hepatic cholesterol and triglyceride concentration compared to the CS group but significantly increased plasma total cholesterol and triglyceride concentration and fecal lipids with a simultaneous increase in HDL-cholesterol level, apo A-I, and the ratio of HDL-cholesterol to total cholesterol (HTR). Reduction of hepatic lipid levels by inclusion of LC intake was observed alongside modulation of hepatic enzyme activities related to cholesterol esterification, fatty acid metabolism and fecal lipids level compared to the CS group. The differential effects of LC intake on the plasma and hepatic lipid profile seemed to be partly due to the fatty acid composition of LC which contains higher MUFA, PUFA and SFA content as well as lower content of *trans *fatty acids compared to CS.

**Conclusions:**

We suggest that LC may exert a dual effect on plasma and hepatic lipid metabolism in an atherogenic animal model. Accordingly, LC, supplemented at 10% in diet, had an anti-atherogenic effect on these *apo E*^*-/- *^mice, and increased fecal lipids, decreased hepatic steatosis, but elevated plasma lipids. Further studies are needed to verify the exact mode of action regarding the complex physiological changes and alteration in lipid metabolism caused by LC.

## Introduction

Fat in foods has great palatability and can be addictive to animals [1,2] and human beings [3]. Fats and oils as they exist in nature must be processed before they are suitable for human consumption [4]. Partial hydrogenation of vegetable oils is a common technological aid to food processing with few or no undesirable effects although it can alter the composition of fatty acids. Types of polyunsaturated fatty acids (PUFA) in dietary fats have been shown to profoundly influence lipid metabolism and physiological function. In particular, linoleic acid (18:2, n-6, LA) and α-linolenic acid (18:3, n-3) are metabolized to highly unsaturated fatty acids which have distinct physiological functions [5]. However, epidemiological evidence suggests a strong relationship between the intake of *trans *fatty acids, which are formed during the hydrogenation process, and the risk of coronary heart disease (CHD) [6-8]. The effect of *trans *fatty acids on the serum lipoprotein profile is at least as unfavorable as that of cholesterol-raising saturated fatty acids, because *trans *fatty acids not only raise low-density lipoprotein (LDL) cholesterol levels but also lower high-density lipoprotein (HDL) cholesterol levels. Later studies, involving either partially hydrogenated fat as the source of *trans *fatty acids or specifically synthesized fat differing in only a single fatty acid, further demonstrate the blood cholesterol raising effects of *trans *fatty acids [9-12].

In this study, we attempted to synthesize a novel structured lipid using corn oil. LA is the primary (in terms of mass consumed) essential fatty acid in corn oil. It can reduce plasma total cholesterol and LDL-cholesterol [13,14], each of which is an established CHD risk factor. In the present study, the novel structured lipid was produced from corn oil by lipase-catalyzed glycerolysis, which produced a lipid rich in monounsaturated fatty acid (MUFA) and PUFA compared to *trans *fats. This novel structured lipid may provide a beneficial effect on lipid metabolism. Accordingly, we examined the physiological effects of our novel structured lipid on the plasma and hepatic lipid profile in *apo E*^*-/- *^mice, an animal model of atherosclerosis.

## Materials and methods

### Test Oil

A commercial shortening (CS, New Orleans, USA) and a low *trans *structured fat from corn oil (LC, Seoul, Korea) were used in this study. 500 g oil blends of anhydrous butterfat (ABF), palm stearin (PS) and corn oil (CO) (8/6/6 and 6/6/9, w/w/w) were added respectively into a 1-L tank stirred-batch type reactor. The tank height and diameter was 11 cm and 15 cm respectively. The enzyme load was 10% of total substrates (50 g, w/w). The reaction was stirred at 230 rpm by an impeller (blade length: 9 cm; blade width: 3.3 cm) and stirrer motor (M Tops MS-3060D, Korea). Lipozyme RM IM and Novozyme 435 were used for blending mixture of 8/6/6 and 6/6/9 by mass (ABF/PS/CO). The reaction for production of the modified-butterfat was carried out for 24 hr in a solvent-free system. Samples were withdrawn during the reaction according to the time course, and then filtered by PTFE syringe membrane filter (25 mm, 0.2 μm, Whatman, USA) to remove the enzyme for analysis. The fatty acid profile was analyzed for each fat used (Table 1).

**Table 1:** Fatty acid composition of two dietary lipids, a hydrogenated *trans *fat and a corn-oil based low *trans *structured fat, used in animal experiment.

(unit : %)		
	CS	LC
14:00	0.11	0.71
16:00	13.42	35.81
16:01	0.07	0.07
18:00	11.56	15.65
18:01	12.64	24.07
18:02	5.39	22.72
18:03	0.22	0.55
20:00	0.06	-
20:01	0.25	-
18:1*T*	43.84	-
18:2*T*	11.91	-
18:3*T*	0.50	0.42
Total *Trans*	56.25	0.42
n-6	5.39	22.72
n-3	0.22	0.55
n-6/n-3 ratio	24.50	41.31
SFA	25.15	52.17
MUFA	12.71	24.14
PUFA	5.61	23.27
P/S	0.22	0.45

SFA, saturated fatty acid; MUFA, monounsaturated fatty acid; PUFA, polyunsaturated fatty acid; P/S, PUFA/SFA ratio.

CS, commercial shortening; LC, low *trans *structured fat from corn oil

### Animals and Diets

Twenty male apolipoprotein E-deficient (*apo E*^*-/-*^) mice, a well established animal model of atherosclerosis, were imported from Jackson Laboratory (Bar Harbor, Maine, USA) at 5 weeks of age. All the mice were individually housed under a constant temperature (24°C) with 12 h light/12 h dark cycle and fed a palletized commercial chow diet for 1 week after arrival. The mice were then randomly divided into two groups (n = 10) and fed the AIN-76 (American Institute of Nutrition, 1977) basal diet containing 10% (wt/wt) test oil for 12 weeks (Table 2).

**Table 2:** Composition of experimental diets composed of a hydrogenated *trans *fat and a corn-oil based low *trans *structured fat (unit : % of diet).

Groups Ingredients	CS	LC
Casein	20	20
Methionine	0.3	0.3
Sucrose	50	50
Cone starch	10	10
Fiber	5	5
Test oils	10	10
Cholinebitartrate	0.2	0.2
Mineral mixture^a^	3.5	3.5
Vitamin mixture^b^	1	1

Total	100	100

^a^AIN-76 mineral mixture (Harlan Teklad Co., Madison, WI).

^b^AIN-76 vitamin mixture (Harlan Teklad Co.).

CS, commercial shortening; LC, low *trans *structured fat from corn oil

Free access was given to food and water. Blood was taken from a tail vein for the determination of plasma total cholesterol and triglyceride concentration at 0, 3rd, 6th, 9th and 12th wks. Every day for the last 4 days, the feces were collected and analyzed for fecal lipids. The food consumption and body weight were measured daily and weekly. At the end of the experimental period, the mice were anesthetized with ketamine-HCl after food was withheld for 14 hours. Blood samples were taken from the inferior vena cava for plasma lipid analysis. The organs and adipose tissues were removed and rinsed with physiological saline. All samples were stored at -70°C until analysis. This experimental design was approved by the Ethics Committee of Kyungpook National University for the care and use of laboratory animals.

### Plasma, hepatic and fecal lipid analysis

The plasma triglyceride, total cholesterol and HDL-cholesterol concentrations were enzymatically determined using a commercial kit (Sigma). The plasma apolipoprotein A-I (apo A-I), apolipoprotein B (apo B) and non-esterified fatty acid (NEFA) were also measured using commercial assay kits (Nitto Boseki Co. Ltd, Japan, NEFA-Wako Pure Chemical Industries). The hepatic lipids were extracted using the procedure developed by Folch et al. [15]. The dried lipid residues were dissolved in 1 ml of ethanol for cholesterol and triglyceride assays. Triton X-100 and a sodium cholate solution (in distilled H_2_O) were added to 200 μL of the dissolved lipid solution to produce final concentrations of 5 g/L and 3 mmol/L, respectively. The hepatic cholesterol and triglycerides were analyzed with the same enzymatic kit as used in the plasma analysis.

The feces from each group were collected daily for 1 week and analyzed for lipids, as described previously with a slight modification [15]. Briefly, the feces were dried and extracted in ice-cold chloroform and methanol (2:1, v/v) for 24 h at 4°C. After centrifugation at 900 × *g *for 10 min, the supernatant was collected, dried at 50°C, and dissolved with ethanol. The fecal cholesterol and triglyceride levels were estimated using the same method as used for the liver.

### Preparation of hepatic tissue enzyme source

Enzyme source fractions from hepatic tissue were prepared as follows. The tissue 0.5 g was homogenized in a fivefold volume of 0.25 M sucrose buffer and centrifuged at 600 × g for 10 min to remove any cell debris and then the supernatant was centrifuged at 10,000 × g for 20 min to isolate the mitochondrial pellet. Finally, the supernatant was further ultracentrifuged at 105,000 × g for 60 min to obtain the cytosol supernatant. Protein contents in the mitochondrial and cytosolic fractions were measured according to the method of Bradford [16] using bovine serum albumin as the standard.

### Hepatic lipid regulating enzyme activities

Glucose-6-phosphate dehydrogenase (G6PD) activity was assayed by spectrophotometric methods according to the procedures described by Pitkanen et al. [17], where the activity was expressed as the reduced NADPH (nmol/min/mg protein). The supernatant cytosolic fractions were analyzed for malic enzyme (ME) (EC 1.1.1.40) using modification of the methods of Ochoa [18]. ME activity was expressed as nmol NADPH (nmol/min/mg protein). The hepatic β-oxidation was measured spectrophtometrically by monitoring the reduction of NAD to NADH in the presence of palmitoyl-CoA [19]. The reaction was initiated by addition of the enzyme source to a reaction cuvette containing 20 mM NAD, 0.33 M dithiothreitol, 1.5% bovine serum albumin, 2% Triton X-100, 10 mM CoA, 1 mM FAD, 100 mM KCN, and 5 mM palmitoyl-CoA in 50 mM Tris-HCl (pH 8.0). The reaction compartment was thermostatted at 37°C, and the reaction rate was followed at 340 nm. Data were expressed as nmol of NAD reduced/min/mg of protein. The carnitine palmitoyl transferase (CPT) activity was determined according to the method of Markwell et al. [20]. The results were expressed as nmol/min/mg protein.

### 3-Hydroxy-3-methylglutaryl (HMG)-CoA reductase and acyl-CoA:cholesterol acyltransferase (ACAT) activities

The HMG-CoA reductase activity in the microsomal fraction was measured with [^14^C]HMG-CoA as the substrate based on a modified method of Shapiro [21], where the activity was expressed as the synthesized mevalonate (pmol/min/mg protein). The ACAT activity in the microsomes was determined by the rate of incorporation of [^14^C]oleoyl-CoA into the cholesterol ester fraction, as described by Erickson [22], where the activity was expressed as synthesized cholesteryl oleate (pmol/min/mg protein).

### Hepatic Tissue Morphology

Livers were removed and fixed in a buffer solution of 10% formaldehyde. Fixed tissues were processed routinely for paraffin embedding, and 4-μm sections were prepared and dyed with hematoxylin-eosin. Stained areas were viewed using an optical microscope × 100 magnification.

### Statistical analysis

All data are presented as the mean ± S.E. Statistically significant differences between the groups were determined with a Student's *t *test (*p *< .05) using the standard social science statistical package (SPSS, Chicago, IL, USA).

## Results

### Food intake, weight gain, FER and organ weights

Weight gain and food efficiency ratio (FER) were significantly higher in the LC group compared to the CS group, with no change in food intake. The weight of liver and heart (g/100 g body weight) was significantly lower in the LC group than in the CS group (Table 3).

**Table 3:** Effect of supplementation of a hydrogenated *trans *fat and a corn-oil based low *trans *structured fat on food intake, weight gain, food efficiency ratio (FER) and organ weights in *apo E*^-^^*/*^^- ^mice.

Group	CS	LC
Food Intake (g/day)	4.32 ± 0.09	4.35 ± 0.11
Weight gain (g/day)	0.12 ± 0.01	0.15 ± 0.01**
FER	0.03 ± 0.00	0.03 ± 0.00*

Organ Weights (g/100 g B.W.)		
Liver	6.78 ± 0.29	5.21 ± 0.08***
Heart	0.46 ± 0.01	0.37 ± 0.02**

Data are mean ± S.E. (*n *= 10).

Values are significantly different from the CS group according to Student's *t *test: *P < .05, **P < .01, ***P < .001.

FER : Food efficiency ratio = body weight gain/food intake; B.W., Body Weight

CS, commercial shortening; LC, low *trans *structured fat from corn oil

### Plasma, hepatic and fecal lipids

The plasma total cholesterol and triglyceride levels were significantly higher in the LC group (Table 4). While free fatty acid concentration was unchanged. The HDL-cholesterol, apo A-I, fecal lipids concentrations and the ratio of HDL-cholesterol to total cholesterol were also significantly higher in the LC group compared to the CS group (Table 4). In addition, hepatic triglyceride, hepatic cholesterol, apo B concentration and atherogenic index, which are all significant risk factors of coronary heart disease, were significantly lower in the LC group (Table 4).

**Table 4:** Effect of supplementation of a hydrogenated *trans *fat and a corn-oil based low *trans *structured fat on plasma, hepatic and fecal lipid levels in *apo E*^-^^*/*^^- ^mice.

	CS	LC
Plasma lipids		
Free fatty acid (mmol/L)	1.14 ± 0.03	1.11 ± 0.04
Triglyceride (mmol/L)	1.46 ± 0.09	2.09 ± 0.08***
Total-C (mmol/L)	16.45 ± 0.81	18.75 ± 0.37*
HDL-C (mmol/L)	0.11 ± 0.02	0.81 ± 0.06***
HDL-C/Total-C (%)	0.60 ± 0.06	4.06 ± 0.39***
Atherogenic index^a^	174.95 ± 18.05	23.39 ± 1.99***
Apo A-I (mg/dL)	4.46 ± 0.57	7.47 ± 0.25***
Apo B (mg/dL)	21.00 ±0.54	17.19 ± 0.60***
Hepatic lipids		
Cholesterol (mmol/g liver)	7.88 ± 0.03	5.83 ± 0.19***
Triglyceride (mmol/g liver)	7.21 ± 0.34	5.27 ± 0.18**
Fecal lipids		
Cholesterol (μmol/g)	4.60 ± 0.28	6.44 ± 0.37**
Triglyceride (μmol/g)	2.22 ± 0.54	4.44 ± 0.85*

Data are mean ± S.E. (n = 10). Values are significantly different from the CS group according to Student's *t *test: *P < .05, **P < .01, ***P < .001. C:cholesterol.

^a ^Atherogenic index: (Total-C-HDL-C)/HDL-C

CS, commercial shortening; LC, low *trans *structured fat from corn oil

### Enzyme activities related to fatty acid metabolism in liver

The hepatic and adipocyte G6PD and ME activity were significantly lower in the LC group compared to the CS groups (Table 5). The activity of hepatic β-oxidation and CPT, the rate-limiting enzyme in fatty acid oxidation, was significantly higher in the LC group compared to the CS group (Table 5).

**Table 5:** Effect of supplementation of a hydrogenated *trans *fat and a corn-oil based low *trans *structured fat on the enzyme activities related to the hepatic fatty acid metabolism in *apo E*^-^^*/*^^- ^mice.

	CS	LC
Fatty acid synthesis		
G6PD (nmol/min/mg protein)	24.90 ± 2.74	7.42 ± 0.30^***^
ME (nmol/min/mg protein)	217.4 ± 18.64	126.58 ± 15.32^**^
Fatty acid oxidation		
β-oxidation (μmol/min/mg protein)	0.43 ± 0.08	5.42 ± 1.13^*^
CPT (nmol/min/mg protein)	19.86 ± 1.42	27.09 ± 1.56^**^

Data are mean ± S.E. (*n *= 10). Values are significantly different from the CS group according to Student's *t *test: *P < .05, **P < .01, ***P < .001. G6PD, glucose-6-phosphate dehydrogenase; ME, malic enzyme; CPT, carnitine palmitoyl transferase.

CS, commercial shortening; LC, low *trans *structured fat from corn oil

### Hepatic HMG-CoA reductase and ACAT activities

**Figure 1:**
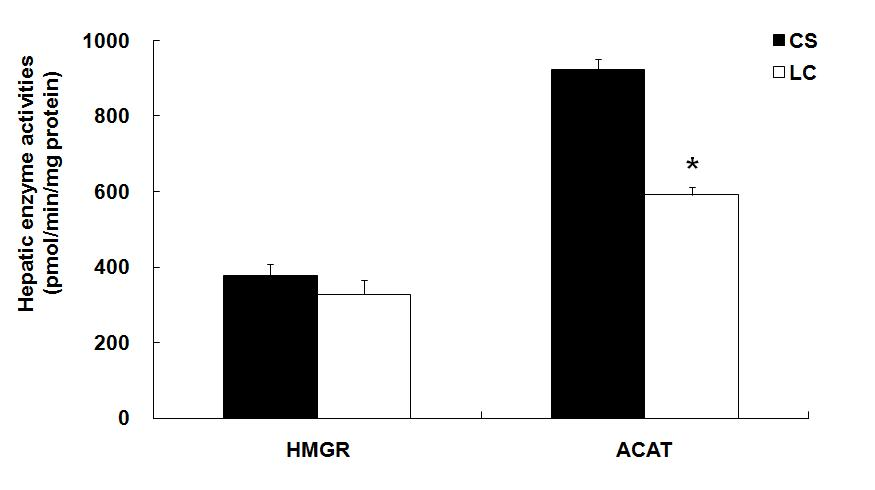
**Effect of supplementation of a hydrogenated *trans *fat and a corn-oil based low *trans *structured fat on HMG-CoA reductase (HMGR) and ACAT activities in *apo E***^***-/-***^** mice : CS group (■) and LC group (□)**. The results represent mean ± S.E. (*n *= 10). Values are significantly different from the CS group according to Student's *t *test: *P < .05. HMGR, 3-hydroxy-3-methylglutaryl-coenzyme reductase; ACAT, acyl CoA: cholesterol acyltransferase. CS, commercial shortening; LC, low *trans *structured fat from corn oil

The hepatic ACAT activity was significantly lower in the LC supplemented group than in the CS group, while the hepatic HMG-CoA reductase activity was not significantly between the groups (Figure 1).

### Morphological changes in hepatocytes

**Figure 2:**
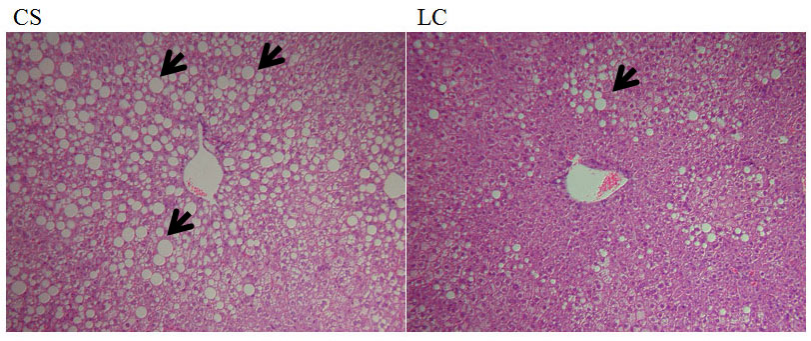
**Effect of supplementation of a hydrogenated *trans *fat and a corn-oil based low *trans *structured fat on hepatic tissue morphology in *apo E*^*-/- *^mice**. The results represent mean ± S.E. (*n *= 10). The results represent mean ± S.E. (*n *= 10). Original magnification × 100. Fat accumulation, indicated by the arrowheads, in the form of large fat droplet is present in liver. CS, commercial shortening; LC, low *trans *structured fat from corn oil

Lipid droplet accumulation in liver was lower in the LC group compared to the CS group when observed in the light microscope (Figure 2). This result was consistent with the hepatic triglyceride and cholesterol profile shown in Table 4.

## Discussion

The present study demonstrates the differential effect of two solid fats, a low *trans *structured fat and a hydrogenated *trans *fat, on plasma and hepatic lipid metabolism in *apo E*^*-/- *^mice. *Trans *fatty acid intake has been convincingly shown to be associated with a significantly higher risk of heart disease based on large epidemiology and clinical studies [23-25]. Previously in a comprehensive review of past studies summarized *trans *fatty acid intake significantly effects blood lipids, in particular the LDL-cholesterol/HDL-cholesterol ratio and total cholesterol/HDL-cholesterol ratio, leading to increased risk of CHD risk [26]. In the present study, plasma HDL-cholesterol, apo A-I concentrations and HTR were significantly increased, whereas apo B level were significantly lower in the LC group than in the CS group. Apo B and apo A-I are thought to be better predictors of CHD risk than total cholesterol and LDL-cholesterol [27]. Apo A-I also acts as a cofactor for lecithin: cholesterol acyltransferase (LCAT) [28], which is an important enzyme involved in removing excess cholesterol from tissues and incorporating it into HDL for reverse cholesterol transport to the liver [29]. Apo B is synthesized in the liver and is present in LDL, IDL and VLDL particles [29], and therefore the total apo B concentration indicates the amount of potentially atherogenic lipoproteins in plasma or liver [30].

Previous studies have shown that eating partially hydrogenated lipids results in an increase in liver phospholipid concentrations [31]. In the present study, hepatic cholesterol, triglyceride and lipid droplet accumulation in liver was also significantly lowered in the LC group than in the CS group which may contribute reducing liver weight. Hence LC consumption appears to prevent the development of hepatic steatosis in apo E deficient mice. Elevated excretion of cholesterol and triglyceride was also observed in LC fed mice. However, plasma total-C and triglyceride concentration were significantly higher in the LC group than in the CS group. It is plausible that the difference in fatty acid composition between CS and LC could have led to the paradoxical finding of an anti-arterogenic effect in liver but negative pro-arteriogenic effect in blood although at present the mechanism is unclear. A commercial low *trans *fat with high MUFA, myristic acid and palmitic acid content also exhibited the same hepatic lipid-lowering effect but paradosical increase in the plasma cholesterol concentration [32]. Current findings support the dual effects of low *trans *fats, in part, can be modulated by the fatty compositions of these structured fats. Saturated fatty acids (SFAs) are major dietary constituents that can raise plasma total-C concentration [33]. The cholesterol raising properties of SFAs can be primarily attributed to myristic acid (14:0) and palmitic acid (16:0). These SFAs may have different effects on serum total cholesterol concentrations [34]. Replacing SFA with MUFA reduces total cholesterol, LDL-cholesterol, and triglyceride concentrations [35]. Alternatively, the differential effects of low *trans *fat on lowering hepatic lipids in *apo E*^*-/- *^mice can be attributed to their specific fatty acid composition. The CS contains more *trans *fatty acid and less SFA per se in comparison to LC which is rich in linoleic acid, however in MUFA and PUFA vice versa is true and the relative amount of SFA is higher.

Regarding cholesterol metabolism, dietary LC did not significantly lower hepatic HMG-CoA reductase activity, however LC significantly decreased hepatic ACAT activity. HMG-CoA reductase is the rate-limiting enzyme in the cholesterol biosynthetic pathway that converts HMG-CoA to mevalonate [36,37]. Intracellular cholesteryl ester (CE) synthesis catalyzed by ACAT serves to store cholesterol in cytosolic droplets and also participates in the hepatic secretion of lipoproteins containing apo B [38-40]. Moreover, ACAT is believed to be involved in cholesterol absorption from the intestine [41] although this was beyond the scope of the present study. Under pathological conditions, the chronic accumulation of CE produced by ACAT in macrophages and arterial smooth muscle cells leads to the characteristic foam cell formation in atherosclerosis [42]. Therefore, ACAT inhibitors are also expected to be cholesterol-lowering and hence potential anti-atherosclerotic agents.

In summary, an increased hepatic fatty acid synthesis was correlated with the availability of fatty acids for triglyceride synthesis [43]. The activities of two hepatic enzymes G6PD and ME, related to fatty acid synthesis, were lower in the LC supplemented *apo E*^*-/- *^mice in this study. Taken together these findings suggest that the LC supplement had a hepatic triglyceride-lowering effect via lowering the hepatic lipogenic enzyme activities while elevating the hepatic CPT and β-oxidation activity in the *apo E*^*-/- *^mice.

## Conclusions

In conclusion, this study provides evidence that low *trans *structured fat from corn oil reduces hepatic lipid accumulation via inhibition of lipogenesis while elevating fatty acid oxidation in *apo E*^*-/- *^mice, a model for atherosclerosis. The differential effects of LC observed on the plasma and liver lipid profile seem to be partly due to the fatty acid composition of LC compared to CS, as LC is high in SFA, MUFA and PUFA content. High SFA in LC may explain the unfavorable effect of LC on plasma triglyceride concentration. We suggest that LC exerts both an anti-atherosclerotic and pro-atherosclerotic effect on plasma and liver lipid metabolism in an atherogenic susceptible animal model. However, further studies are required to verify differential the effects of specific fatty acid composition in low *trans *fats, on lipid metabolism, to justify recommendations to use low *trans *fats such as LC in food processing.

## Competing interests

The authors declare that they have no competing interests.

## Authors' contributions

CYY, KEY, KHJ, JSM, LKT and CMS participated in the design of the study, sample collection, analysis, statistical analysis, and writing of this paper. All authors read and approved the final manuscript.

## References

[B1] Imaizumi M Takeda M Fushiki T Effects of oil intake in the conditioned place preference test in mice Brain Res 2000 870 150 6 10.1016/S0006-8993(00)02416-1 10869512

[B2] Takeda M Sawano S Imaizumi M Fushiki T Preference for corn oil in olfactory-blocked mice in the conditioned place preference test and the two-bottle choice test Life Sci 2001 69 847 54 10.1016/S0024-3205(01)01180-8 11487096

[B3] Kuller LH Nutrition, lipids, and cardiovascular disease Nutr Rev 2006 64 S15 26 10.1111/j.1753-4887.2006.tb00230.x 16532896

[B4] González C Resa JM Concha RG Goenaga JM Enthalpies of mixing and heat capacities of mixtures containing acetates and ketones with corn oil at 25°C J Food Eng 2007 79 1104 9

[B5] Kaku S Ohkura K Yunoki S Nonaka M Tachibana H Sugano M Yamada K Dietary gamma-linolenic acid dose-dependently modifies fatty acid composition and immune parameters in rats Prostaglandins Leukot Essent Fatty Acids 2001 65 205 10 10.1054/plef.2001.0312 11728173

[B6] Samman S Dietary trans fatty acids and coronary heart disease Food Australia 1995 47 S10 3

[B7] Khosla P Hayes K Dietary trans-monounsatyrated fatty acids negatively impact plasma lipids in humans: critical review of the evidence J Am Coll Nutr 1996 15 25 39 10.1080/07315724.1996.10718607 8829088

[B8] Ascherio A Willett WC Health effects of trans fatty acids Am J Clin Nutr 1997 66 1006S 10S 9322581 10.1093/ajcn/66.4.1006S

[B9] Nestel P Noakes M Belling B McArthur R Clifton P Janus E Abbey M Plasma lipoprotein lipid and Lp[a] changes with substitution of elaidic acid for oleic acid in the diet J Lipid Res 1992 33 1029 36 1431582

[B10] Wood R Kubena K O'Brien B Tseng S Martin G Effect of butter, mono- and polyunsaturated fatty acid-enriched butter, trans fatty acid margarine and zero trans fatty acid margarine on serum lipids and lipoproteins in healthy men J Lipid Rrs 1993 34 1 11 8445333

[B11] Lichtenstein AH Ausman LM Carrasco W Ordovas JM Schaefer EJ Hydrogenation impairs the hypolipidemic effect of corn oil in humans Arterioscler Thromb 1993 13 154 61 8427852 10.1161/01.atv.13.2.154

[B12] Seppanen-Laakso T Vanhanen H Laakso I Kohtamaki H Viikari J Replacement of margarine on bread by rapeseed and olive oils: effects on plasma fatty acid composition and serum cholesterol Ann Nutr Metab 1993 37 161 74 10.1159/000177765 8105749

[B13] Hayes KC Dietary fatty acids, cholesterol, and the lipoprotein profile Br J Nutr 2000 84 397 9 11103209

[B14] Mensink RP Zock PL Kester AD Katan MB Effects of dietary fatty acids and carbohydrates on the ratio of serum total to HDL cholesterol and on serum lipids and apolipoproteins: a meta-analysis of 60 controlled trials Am J Clin Nutr 2003 77 1146 55 12716665 10.1093/ajcn/77.5.1146

[B15] Folch J Lees M Sloan-Stanley GH A simple method for the isolation and purification of total lipids from animal tissues J Biol chem 1957 226 497 509 13428781

[B16] Bradford MM A rapid and sensitive method for the quantitation of microgram quantities of protein utilizing the principle of protein-dye binding Anal Biochem 1976 72 248 54 10.1016/0003-2697(76)90527-3 942051

[B17] Pitkanen E Pitkanen O Uotila L Enzymatic determination of unbound d-mannose in serum Eur J Clin Chem Clin Biochem 1997 35 761 6 9368794 10.1515/cclm.1997.35.10.761

[B18] Ochoa S Colowick SP, Kaplan NO Malic enzyme: malic enzymes from pigeon and wheat germ Methods in Enzymology 1995 1 Academic Press, New York, NY 323 6

[B19] Lazarow PB Assay of peroxisomal β-oxidation of fatty acids Methods Enzymol 1981 72 315 9 full_text 7031421 10.1016/s0076-6879(81)72021-4

[B20] Markwell MAK McGroarty EJ Bieber LL Tolbert NE The subcellular distribution of carnitine acyltransferases in mammalian liver and kidney J Biol Chem 1973 248 3426 32 4702872

[B21] Shapiro DJ Nordstrom JL Mitschelen JJ Rodwell VW Schimke RT Micro assay for 3-hydroxy-3-methylglutaryl-CoA reductase in rat liver and in L-cell fibroblasts Biochim Biophys Acta 1974 370 369 77 4441486 10.1016/0005-2744(74)90098-9

[B22] Erickson SK Shrewsbury MA Brooks C Meyer DJ Rat liver acyl-coenzyme A:cholesterol acyltransferase: its regulation in vivo and some of its properties in vitro J Lipid Res 1980 21 930 41 7441061

[B23] Mensunk RP Zock PL Kester ADM Katan MB Effects of dietary fatty acids and carbohydrates on the ration of serum total to HDL cholesterol and on 60 controlled trials Am J Clin Nutr 2003 77 1146 55 12716665 10.1093/ajcn/77.5.1146

[B24] Stender S Dyerber J Influence of trans fatty acids on health Nutr Metab 2004 48 61 6 10.1159/000075591 14679314

[B25] Mathan NR Welty FK Barret HR Harusz C Dolnikowski GG Parks JS Eckel RH Schaefer EJ Lichtenstein AH Dietary hydrogenated fat increases high-density lipoprotein apoA-I catabolism and decreases low-density lipoprotein apoB-100 catabolism in hypercholesterolemic women Arterioscler Thromb Vasc Biol 2004 24 1092 7 10.1161/01.ATV.0000128410.23161.be 15087307

[B26] Ascherio A Katan MB Zock PL Stampfer MJ Willett WC Trans fatty acids and coronary heart disease N Engl J Med 1999 340 1994 8 10.1056/NEJM199906243402511 10379026

[B27] Walldius G Jungner I Holme I Aastveit AH Kolar W Steiner E High apolipoprotein B, low apolipoprotein A-I, and improvement in the prediction of fatal myocardial infarction (AMORIS study): a prospective study Lancet 2001 358 2026 33 10.1016/S0140-6736(01)07098-2 11755609

[B28] Phillips MC Gillotte KL Haynes MP Johnson WJ Lund-Katz S Rothblat GH Mechanisms of high density lipoprotein-mediated efflux of cholesterol from cell plasma membranes Atherosclerosis 1998 137 S13 7 10.1016/S0021-9150(97)00312-2 9694536

[B29] Betteridge DJ Morrell JM Clinicians Guide to Lipids and Coronary Heart Disease 1999 London

[B30] Genest J Jr The measurement of apolipoprotein B should replace the conventional lipid profile in screening for cardiovascular risk. I beg to differ Can J Cardiol 1992 8 138 40 1559190

[B31] Koga T Yamato T Ikeda I Sugano M Effects of randomization of partially hydrogenated corn oil on fatty acid and cholesterol absorption, and tissue lipid levels in rats Lipids 1995 30 935 40 10.1007/BF02537485 8538381

[B32] Cho YY Kwon EY Kim HJ Park YB Lee KT Park T Choi MS Low trans structured fat from flaxseed oil improves plasma and hepatic lipid metabolism in *apo E(-/-) *mice Food Chem Toxicol 2009 47 1550 5 10.1016/j.fct.2009.04.001 19361550

[B33] Grundy S Denke M Dietary influences on serum lipids and lipoproteins J Lipid Res 1990 31 1149 72 2205699

[B34] Mensink RP Effects of the individual saturated fatty acids on serum lipids and lipoprotein concentrations Am J Clin Nutr 1993 57 711S 4S 8475888 10.1093/ajcn/57.5.711S

[B35] Mensink RP Zock PL Kester AD Katan MB Effects of dietary fatty acids and carbohydrates on the ratio of serum total to HDL cholesterol and on serum lipids and apolipoproteins: A meta-analysis of 60 controlled trials Am J Clin Nutr 2003 77 1146 55 12716665 10.1093/ajcn/77.5.1146

[B36] El-Sohemy A Kendall CW Rao AV Archer MC Bruce WR Dietary cholesterol inhibits the development of aberrant crypt foci in the colon Nutr Cancer 1996 25 111 7 10.1080/01635589609514433 8710680

[B37] El-Sohemy A Bruce WR Archer MC Inhibition of rat mammary tumorigenesis by dietary cholesterol Carcinogenesis 1996 17 159 62 10.1093/carcin/17.1.159 8565127

[B38] Yao Z McLeod RS Synthesis and secretion of hepatic apolipoprotein B-containing lipoproteins Biochim Biophys Acta 1994 1212 152 66 8180241 10.1016/0005-2760(94)90249-6

[B39] Sniderman AD Cianflone K Substrate delivery as a determinant of hepatic apoB secretion Arterioscler Thromb 1993 13 629 36 8485114 10.1161/01.atv.13.5.629

[B40] Thompson GR Naoumova RP Watts GF Role of cholesterol in regulating apolipoprotein B secretion by the liver J Lipid Res 1996 37 439 47 8728309

[B41] Chang CC Sakashita N Ornvold K Lee O Chang ET Dong R Lin S Lee CY Strom SC Kashyap R Fung JJ Farese RV Jr Patoiseau JF Delhon A Chang TY Immunological quantitation and localization of ACAT-1 and ACAT-2 in human liver and small intestine J Biol Chem 2000 275 28083 92 10846185 10.1074/jbc.M003927200

[B42] Brown MS Goldstein JL Lipoprotein metabolism in the macrophage: implications for cholesterol deposition in atherosclerosis Annu Rev Biochem 1983 52 223 61 10.1146/annurev.bi.52.070183.001255 6311077

[B43] Memon RA Grunfeld C Moser AH Feingold KR Fatty acid synthesis in obese insulin resistant diabetic mice Horm Metab Res 1994 26 85 7 10.1055/s-2007-1000778 8200619

